# Occupational therapy in Australian residential aged care facilities: A systematic mapping review

**DOI:** 10.1111/1440-1630.12824

**Published:** 2022-06-17

**Authors:** Lora Calderone, Michelle Bissett, Matthew Molineux

**Affiliations:** ^1^ Discipline of Occupational Therapy, School of Health Sciences and Social Work Griffith University Brisbane Queensland Australia; ^2^ Faculty of Health Southern Cross University Bilinga Queensland Australia

**Keywords:** Australia, mapping review, occupational therapy, older adults, residential aged care, residents

## Abstract

**Introduction:**

Australia's population is ageing, resulting in more older adults living in residential aged care facilities. Occupational therapy scope of practice in Australian residential aged care facilities is significantly influenced by the government funding instrument. As the current government funding instrument is administratively inefficient, insufficiently discriminates between residents' care needs and provides perverse incentives, a new funding model is set to be implemented. This creates an opportunity for a review of the current evidence base to support the lobbying of national associations to shape occupational therapy practice. The research question that guided this systematic mapping review was as follows: What is the current state of scholarship about occupational therapy in Australian residential aged care facilities?

**Methods:**

A search of four databases (CINAHL, Medline, Embase and Scopus) was conducted and 1,617 papers were identified. All papers were screened through a two‐phase process: (i) title and abstract review and (ii) full text review, using pre‐determined inclusion and exclusion criteria to identify papers relevant to this review. A data extraction tool was designed in Microsoft Excel® and was used to extract data from the included papers.

**Results:**

Twelve Australian articles were published between 1986 and 2020, most frequently authored by an occupational therapist. Included articles were classified into four groups: articles including occupational therapists as participants, articles about occupational therapy practice, articles exploring an occupational perspective of residents and articles with limited exploration of occupational therapy.

**Conclusion:**

This review identified that there is a dearth of Australian occupational therapy literature. This creates challenges for occupational therapists seeking evidence to guide their practice to optimise resident health and well‐being and for national associations seeking to lobby for the profession. Consequently, there is a crucial need to develop the evidence base to support the profession within this practice setting and, ultimately, residents.

**Key Points for Occupational Therapy**

1. There is a dearth of literature exploring occupational therapy in Australian RACFs.
2. Future research should explore current and potential occupational therapy practice in Australian residential aged care facilities.
3. Academic‐clinician partnerships, internal to the profession, are recommended to build the evidence base.

## 1 INTRODUCTION

In 2017, 15% of Australians were aged 65 or older (Australian Institute of Health and Welfare, 2018). The Australian Bureau of Statistics (2019) has predicted that this figure will grow steadily due to increased life expectancy and low fertility. An increasing ageing population will result in more older adults living in residential aged care facilities (RACFs) who will receive support in areas including engagement in daily occupations (Australian Bureau of Statistics, 2015).

Occupational therapists have a long history of advancing the health and well‐being of older adults living in RACFs in Australia and internationally (Dancewicz & Bissett, 2020). Although funding and employment models vary across the globe, occupational therapy commonly involves promotion of occupational engagement that can include recommendations for assistive devices and/or proposing adaptations to the environment (Dancewicz & Bissett, 2020). Hammill et al. (2020) argued that occupational therapists within RACFs provide a unique contribution, supporting residents' occupational performance and engagement. Toledano‐González et al. (2018) identified that occupational therapy can improve residents' psychological well‐being and self‐efficacy. Marshall and Mackenzie (2008) emphasised that occupational engagement can contribute to supporting residents to have rich and meaningful lives.

Australian RACFs are funded by the Commonwealth Government (Australian Government Department of Health, 2021). The current funding model, the Aged Care Funding Instrument (ACFI), requires a determination as to a resident's level of care, and this determines the subsidy the RACF receives to provide the required care (Australian Government Department of Health, 2017). The ACFI provides parameters for the scope and service provision of most occupational therapy practice in RACFs (Australian Government Department of Health, 2017; Hamilton & Menezes, 2011), notably chronic pain management through therapeutic massage and electrotherapy (Occupational Therapy Australia, 2019). There is some, less common, occupational therapy service provision that is funded by residential care providers, which is not limited by the ACFI guidelines. Occupational Therapy Australia (2019) argues that the ACFI is restrictive in terms of occupational therapy practice and is in misalignment with the core values of the profession. In response to this misalignment, Occupational Therapy Australia (2021) continues to lobby for change through relevant submissions and correspondence, including to the Royal Commission into Aged Care Quality and Safety. Advocacy includes improving occupational therapy services for older adults in RACFs and ensuring occupational therapists are permitted, and encouraged, to use their unique knowledge and expertise.

Due to the findings that the ACFI is administratively inefficient, insufficiently discriminates between resident's care needs and provides perverse incentives (Eagar et al., 2020), a new funding model is set to be implemented in October 2022 (Australian Government Department of Health, 2021). This provides an opportunity for policymakers to consider the scope of occupational therapy practice in RACFs and positions the need for a timely review of the current evidence base exploring occupational therapy practice in Australian RACFs. Existing literature has the potential to support the lobbying of national associations (e.g., Occupational Therapy Australia and Allied Health Professions Australia) to shape occupational therapy practice under the coming funding model. Therefore, the research question that guided this systematic mapping review was as follows: What is the current state of scholarship about occupational therapy in Australian RACFs?

## 2 METHODS

Systematic mapping reviews involve a comprehensive search of existing literature to describe and categorise the evidence base of interest (Hammick et al., 2010). This includes quantity of literature, research methods employed, topics explored and other key features (Grant & Booth, 2009). From this search, identification of gaps in the existing literature can be made and used to inform further literature reviews or research (Grant & Booth, 2009; Hammick et al., 2010).

### 2.1 Study protocol

The protocol for this study was developed from the approaches detailed in a previous mapping review (Roberts et al., 2020) that was underpinned by the Best Evidence in Medical Education Collaboration (Hammick et al., 2010). The protocol entailed development of a comprehensive search strategy, article selection using inclusion and exclusion criteria, data extraction, and data analysis.

#### 2.1.1 Search strategy

A comprehensive search strategy, developed in collaboration with a health librarian, was conducted in four databases (CINAHL, Medline, Embase and Scopus). The search terms were (i) ‘occupational therap*’ in full text, AND (ii) ‘residential care’ OR ‘residential care facilit*’ OR ‘residential home*’ OR ‘nursing home*’ OR ‘aged care’ OR ‘geriatric care’ OR ‘old age home*’ OR ‘old‐age home*’ OR ‘care home’ OR ‘long term care’ OR ‘long‐term care’ OR ‘long‐term‐care’ OR ‘assisted living’ in title or abstract AND older OR elder* OR aged OR senior* OR geriatric* OR aging OR ageing OR resident* in title or abstract. This search was completed in April 2021 and updated in February 2022 with an additional limit of papers published between 2021 and 2022. This was to ensure this mapping review was current at time of publication. A total of 2,657 articles were retrieved with 1,040 identified as duplicates, leaving 1,617 articles to undergo review.

#### 2.1.2 Data management and article selection

All papers were exported from the databases into EndNote®, a reference management software. EndNote® was used to store and further filter the retrieved papers through application of the inclusion and exclusion criteria. A Microsoft Excel® spreadsheet was created to record the eligibility or ineligibility of the papers. To be included in the review, papers needed to meet the following criteria:

1. Be published in English;
2. Be published in a peer‐reviewed journal;
3. Describe or propose occupational therapy practice in Australia;
4. Describe occupational therapy practice in RACFs OR propose an occupational therapy research study in RACFs OR describe/propose a program within RACFs which included occupational therapy;
5. Describe occupational therapy related to residents living in permanent RACFs;
6. Be a research study OR an opinion piece relating to occupational therapy in RACFs OR present a view about occupational therapy in RACFs from the perspective of another health professional;
7. Contain either older adults OR occupational therapists as all or some of the participants in papers that reported a participant group.

Papers were excluded if they: (i) were published in a language other than English, (ii) were a newsletter article, conference presentation or book chapter, (iii) described or proposed occupational therapy practice in countries other than Australia, (iv) were contexts other than RACFs or permanent places of residence for older adults including, but not limited to, retirement communities and skilled nursing facilities, (v) described or proposed student occupational therapy practice or (vi) were systematic reviews.

**FIGURE 1:**
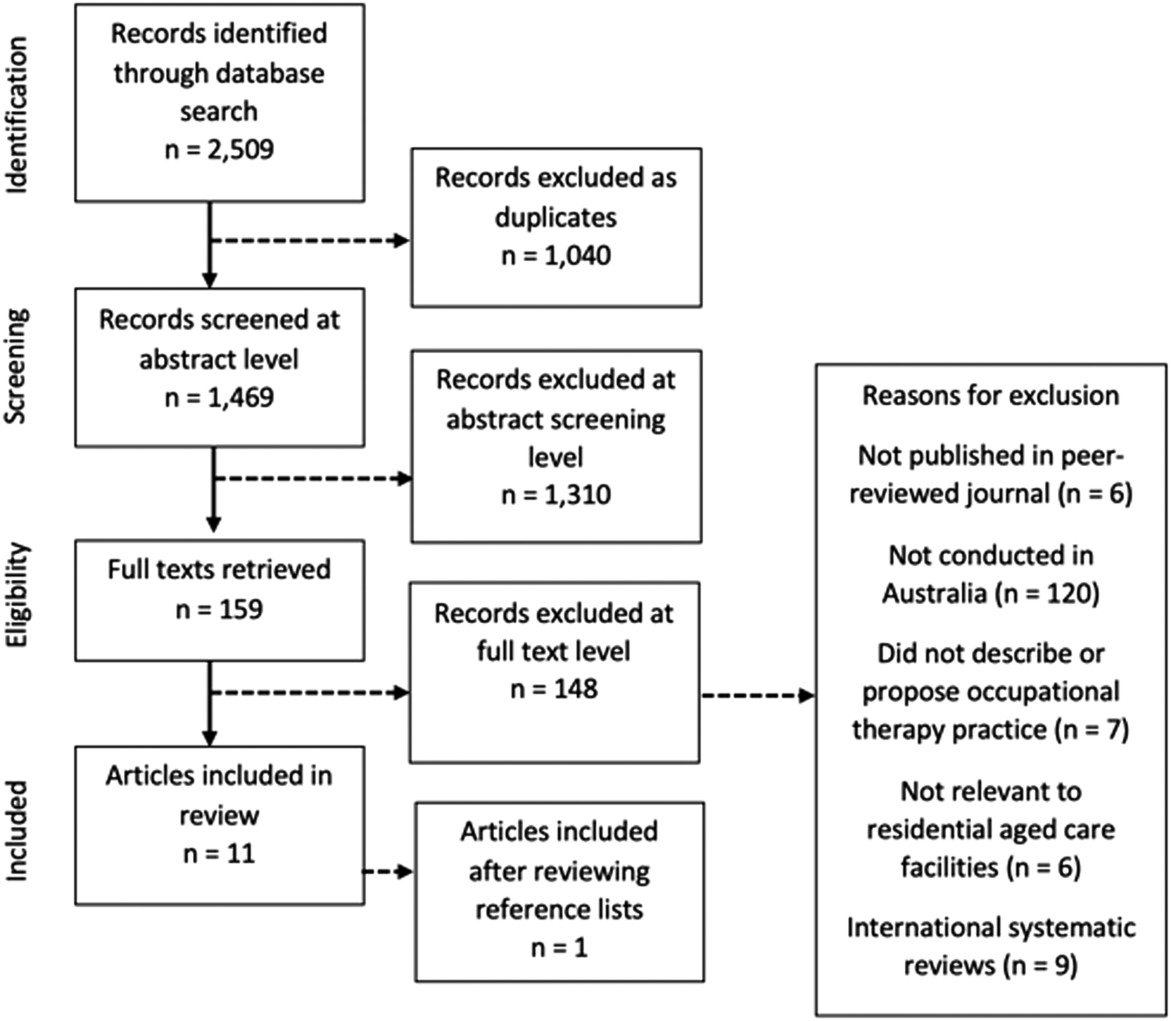
Diagram demonstrating the process of screening and including articles (Moher et al., 2009)

Figure 1 illustrates a diagram adapted from Preferred Reporting Items for Systematic Reviews and Meta‐Analyses (Moher et al., 2009) demonstrating the process of screening and selecting papers for inclusion. Screening of the retrieved papers was completed in two phases: (i) title and abstract review and (ii) full text review. In both phases, this process involved two reviewers independently screening the articles. When the reviewers did not agree, consensus was achieved at a team meeting. In the first phase, 1,617 papers were screened, and 1,458 papers were excluded, leaving 159 papers. After full text review, 11 articles met all seven inclusion criteria and were included in this systematic mapping review. The reference lists of the included articles were reviewed, and one additional paper was identified for inclusion.

#### 2.1.3 Data extraction and analysis

A data extraction tool was created in Microsoft Excel® to collate key information from the included articles such as author/s, year of publication, article title, journal, professional background of author/s, type of publication, study design employed, research aim/s, objective/s or question/s, number of participants included in the study, participant demographic and key findings. The first author extracted data from all included articles. This extraction was then checked by another member of the research team. When discrepancies occurred, they were discussed and resolved during a team meeting.

## 3 RESULTS

### 3.1 Overview of included articles

Twelve articles were included in this systematic mapping review, and an overview of these papers is provided in Table 1. All articles were published between 1986 and 2020, with the majority (75%) published between 2008 and 2015. The articles were published in eight journals, with the largest number in the *Australian Occupational Therapy Journal* (*n* = 3). Ten of the included articles were exclusively set in RACFs, and two (Bennett et al., 2011; Smith, 1986) included data relevant to RACFs. Eight of the articles had a primary author who had a professional background in occupational therapy. The first author of the other four articles were from nursing (Baldacchino & Bonello, 2013a; Baldacchino & Bonello, 2013b), medicine (Loh et al., 2009) and industrial design (Blackler et al., 2018).

**TABLE 1:** Table of articles

Author and year	Journal	Professional background of author/s	Research design as reported by author/s	Setting	Participants	Diagnosis of residents	Aim/objective
Baldacchino and Bonello (2013a)	*British Journal of Nursing*	Nursing	Mixed methods	Exclusively RACFs	Cross‐sectional comparative data: Residents (*n* = 137) including 30 Australian residents; Interviews: Residents (*n* = 42) including 5 Australian residents.	Not specified	Assess the levels and nature of depression and anxiety among Maltese and Australian residents and detect any differences between the cohorts.
Baldacchino and Bonello (2013b)	*British Journal of Nursing*	Nursing	Mixed methods	Exclusively RACFs	Cross‐sectional comparative data: Residents (*n* = 137) including 30 Australian residents; Interviews: Residents (*n* = 42) including 5 Australian residents.	Not specified	Assess the levels and nature of depression and anxiety among Maltese and Australian residents and detect any differences between the cohorts.
Bennett et al. (2011)	*Australian Occupational Therapy Journal*	Occupational therapy	Survey	Includes RACFs	Occupational therapists (*n* = 134) including 45 who worked in RACFs	N/A	Describe Australian occupational therapy practice with people with dementia.
Blackler et al. (2018)	*Sage Open Medicine*	Industrial design; occupational therapy	Audit, interviews and observation	Exclusively RACFs	Residents (*n* = 19) and staff (*n* = 8) including 2 occupational therapists	Absence of cognitive impairment	To provide greater understanding of the context and needs of aged care seating.
Cox et al. (2011)	*British Journal of Occupational Therapy*	Occupational therapy; dietetics; biostatistics	Quasi‐experimental	Exclusively RACF	Residents (*n* = 7)	Alzheimer's disease	Examine the positive effect of live music on affective, functional and leisure behaviour among residents with Alzheimer's disease.
Cox et al. (2014)	*British Journal of Occupational Therapy*	Occupational therapy; dietetics; biostatistics	Quasi‐experimental	Exclusively RACF	Residents (n = 7)	Alzheimer's disease	Investigate whether live music on an individual basis can reduce agitated behaviour among residents with Alzheimer's disease.
Hodges and Schmidt (2009)	*Occupational Therapy International*	Occupational therapy	Phemonemological	Exclusively RACF	Residents (*n* = 4)	Absence of cognitive impairment	To provide post‐war European residents opportunity to document their life experiences in purpose to provide insight into this phenomenon.
Loh et al. (2009)	*Journal of the American Medical Directors Association*	Medicine	Mixed methods	Exclusively RACFs	Survey: Staff (*n* = 53) including 7 allied health; Focus Group: Staff (*n* = 32) Residents (*n* = 12)	Not specified	Determine why introduction of health consulting services via Telehealth video conference consultations failed in RACFs.
Marshall and Mackenzie (2008)	*Australian Occupational Therapy Journal*	Occupational therapy	Phenomenological	Exclusively RACF	Residents (*n* = 11)	Absence of cognitive impairment	Explore resident's experiences of adjusting to hostel care and, consequently, the implications for occupational therapy practice.
Richards et al. (2015)	*Australian Occupational Therapy Journal*	Occupational therapy	Observations and interviews	Exclusively RACF	Residents (*n* = 13) Staff (*n* = 4)	Dementia	Observe and investigate the opportunities for occupational engagement among residents with dementia in traditional vs. non‐traditional rural facilities.
Rosa Hernandez et al. (2020)	*Health & Social Care in the Community*	Occupational therapy	Ethnographic	Exclusively RACF	Observations: Residents (*n* = 12) Caregivers (*n* = 10) Interviews: Residents (*n* = 5) Caregivers (*n* = 5) Staff (*n* = 3)	Not specified	Explore participants experiences of engagement in an intergenerational playgroup.
Smith (1986)	*Physical and Occupational Therapy in Geriatrics*	Occupational therapy		Includes RACFs	N/A	N/A	Not stated.

Abbreviation: RACFs, residential aged care facilities.

Eleven of the articles were research studies and one was a description of practice (Smith, 1986). Several study designs were employed, however, only eight research studies explicitly stated which design was implemented: quantitative (*n* = 2), qualitative (*n* = 3), and mixed methods (*n* = 3). The 11 research studies varied in sample sizes, ranging from four to 137 participants. When only Australian participants (residents and occupational therapists) were included, the sample sizes range reduced to be from four to 45.

Three articles included occupational therapists as participants. One study exclusively included occupational therapists, however, only 34% of the participants worked in RACFs (Bennett et al., 2011). The other two research studies were exclusively set in RACFs, with occupational therapists making up less than 25% of the staff participants (Blackler et al., 2018; Loh et al., 2009).

Ten of the 11 research studies included older adults as participants. Some papers (*n* = 3) excluded residents with a diagnosed cognitive condition or dementia, while other papers (*n* = 3) included these residents. Some articles (*n* = 4) did not use an inclusion or exclusion criteria to select participants based on diagnosis (Baldacchino & Bonello, 2013a; Baldacchino & Bonello, 2013b; Bennett et al., 2011; Rosa Hernandez et al., 2020).

### 3.2 Areas of focus

Analysis of the articles revealed that they could be classified into four distinct groups: articles including occupational therapists as participants, articles about occupational therapy practice, articles exploring an occupational perspective of residents and articles with limited exploration of occupational therapy.

#### 3.2.1 Articles including occupational therapists as participants

Three articles included occupational therapists as participants (Bennett et al., 2011; Blackler et al., 2018; Loh et al., 2009). One was a national survey that included occupational therapists working in RACFs and found that practice in this setting included providing education, assessment, equipment prescription and cognitive and behavioural intervention (Bennett et al., 2011). Key barriers to practice identified in this study included time constraints and role restrictions imposed by RACFs. The study revealed that less than 10% of referrals were open referrals, meaning those that permit occupational therapists to collaboratively identify occupational goals with residents and develop subsequent interventions. Furthermore, 53% of the participants admitted to feeling no to little confidence regarding being up to date with the current evidence base. Blackler et al. (2018) explored seating in RACFs through observations and interviews to understand residents and staff perceptions of seating in the facility. Occupational therapists and residents identified similar positive and negative aspects of chair design. Occupational therapists were valued members of the team who were relied upon to provide seating recommendations throughout the facility. The third study (Loh et al., 2009) aimed to understand the failure of telehealth as a mode of service in RACFs. Occupational therapists were part of multidisciplinary teams who were participants in this study. Limited data were extracted from this article due to very little consideration of occupational therapy.

#### 3.2.2 Articles about occupational therapy practice

Four articles described occupational therapy intervention (Cox et al., 2011; Cox et al., 2014; Hodges & Schmidt, 2009; Smith, 1986). Two of those investigated the effect of live music on residents diagnosed with Alzheimer's disease (Cox et al., 2011; Cox et al., 2014). In these studies, an occupational therapist arranged a live violin recital for residents. The occupational therapist evaluated the outcomes using a standardised measurement: a modified Cohen‐Mansfield Agitation Inventory. One study found that live music significantly increased more than half (56%) of the positive behaviours that were measured, such as smiling and reminiscing conversation (Cox et al., 2011). The other study, which focused on agitated behaviours, found a significant reduction of wandering, restlessness and repetitious manners (Cox et al., 2014). The authors argued that this increased the physical safety of residents through reduction of falls and fatigue (Cox et al., 2014).

Hodges and Schmidt (2009) explored the value of reminiscing and documenting one's life experiences with post‐war European residents. Findings demonstrated that reminiscence enabled residents to reconnect with, validate and express their past. Additionally, residents reported that reminiscing allowed them to feel engaged in the present, as life in a RACF lacked intensity, excitement and diversity of occupations. Smith (1986) described the practice of a multidisciplinary team that provided interventions across three RACFs over 6 months. The team, which included an occupational therapist, provided education on community resources and assistive devices and recommended leisure activities for residents. Smith (1986) reported this intervention resulted in improvements across residents' mobility and emotional statuses.

#### 3.2.3 Articles exploring an occupational perspective of residents

Three articles, authored by occupational therapists, provided insight into residents' occupational engagement (Marshall & Mackenzie, 2008; Richards et al., 2015; Rosa Hernandez et al., 2020). Marshall and Mackenzie (2008) explored the occupational engagement of older adults who had recently transitioned to living in a hostel. The authors interviewed 11 residents and found that eight of them had adjusted positively to the new environment. The findings demonstrated that successful transition was positively impacted by maintaining occupational roles and an occupational identity through attention to personal objects and occupational engagement. Richards et al. (2015) aimed to compare occupational engagement in traditional and non‐traditional RACFs for older adults with dementia. The authors described traditional facilities as ones that align with general guidelines for building design and focus on physical care needs rather than the implementation of social and environmental health promoting strategies. On the other hand, non‐traditional facilities referred to open floor plans, greater outdoor occupational opportunity and minimisation of clinical equipment. From examining these facilities through an occupational lens and conducting interviews with staff and residents, the authors found non‐traditional facilities better engaged residents with dementia in activities such as spontaneous conversation and productive occupations. Non‐traditional facilities provided a more inclusive physical environment and opportunities for spontaneous occupational engagement. This approach responded better to residents' present capabilities and mood. Rosa Hernandez et al. (2020) investigated occupational engagement in an intergenerational play group set within a RACF. Through a combination of observations and interviews, it was found engaging in the group provided residents with opportunities to reminisce, socially interact and build family‐like relationships.

#### 3.2.4 Articles with limited exploration of occupational therapy

Although three articles met the inclusion criteria, they provided little information on Australian occupational therapy practice in RACFs (Baldacchino & Bonello, 2013a; Baldacchino & Bonello, 2013b; Loh et al., 2009). Two of these (Baldacchino & Bonello, 2013a; Baldacchino & Bonello, 2013b) included the same participant group with authors reporting the study's findings across two articles. The authors briefly stated that the current role of occupational therapy within the studied RACFs included coordination of recreational programs such as knitting and crafts. The authors recommended that occupational therapists should aim to maintain residents' mobility. As previously discussed, Loh et al. (2009) included occupational therapists as participants, however, limited data were relevant to occupational therapy practice.

## 4 DISCUSSION

This systematic mapping review identified a dearth of peer‐reviewed publications that explore occupational therapy in Australian RACFs. Additionally, the literature that was found is ‘low’ when considered against published hierarchies of evidence (National Health and Medical Research Council, 2009). As examples, many studies were quasi‐experimental or qualitative in design and not rigorous randomised controlled trials. The absence of high‐quality research examining occupational therapy practice in Australian RACFs is an issue for the profession. The ramifications of this limited evidence base are explored in this section and recommendations for future research are discussed.

The lack of evidence is problematic for national associations who are seeking to lobby for occupational therapy practice at this crucial time. As new models of service delivery are being developed, the profession needs to advocate about the contribution that occupational therapists can make to the lives of residents. At present, advocacy efforts by Occupational Therapy Australia (2021) take place without a strong Australian evidence base and, therefore, are limited to drawing from international literature such as that summarised by Dancewicz and Bissett (2020). This presents challenges when advocating to policymakers to ensure the new funding model permits occupational therapists to use their unique knowledge and expertise to enhance the health of residents. This should be done with consideration of the Aged Care Quality Standards (Australian Government Aged Care Quality and Safety Commission, 2019), which positions older adults at the centre of residential care provision, to ensure that occupational therapy practice is both client centred and evidence based. Moving forward, we encourage occupational therapy researchers in aged care to consider the inclusion of older adults living in RACFs in their research. This will support the development of the evidence base, from which the profession can advocate and lobby for occupational therapy scopes of practice that reflect the core business of occupational therapy.

The limited Australian evidence is surprising given the long history of occupational therapy practice in this setting. A recent scoping review of international literature, which examined scholarship on interventions and outcome measures used by occupational therapists working in RACFs, demonstrated that occupational therapists can work with residents to achieve occupational goals (Dancewicz & Bissett, 2020). Furthermore, Hocking (1997) identified that occupational therapists can support residents to maintain their occupational roles. Similar to the international evidence base, the limited Australian literature identified that providing a diversity of occupational opportunities promotes occupational engagement (Marshall & Mackenzie, 2008; Richards et al., 2015). Additionally, Richards et al. (2015) emphasised that residents have unique occupational identities that must be considered when creating occupational opportunities. However, in comparison with the international evidence base, occupational therapy in Australian RACFs remains largely unexplored. It is recommended further research is conducted to support older adults residing in Australian RACFs to remain occupationally engaged.

While international research provides valuable evidence to support occupational therapy practice, there are challenges regarding its applicability to Australian practice. Building the Australian evidence base is crucial to recognise the influence of uniquely Australian factors, such as the ACFI (Australian Government Department of Health, 2021; Hamilton & Menezes, 2011) and the Aged Care Quality Standards (Australian Government, 2021). These institutional directives significantly influence occupational therapy practice in Australian RACFs as the ACFI currently dictates most occupational therapy practice (Australian Government Department of Health, 2017) and the Quality Standards set expectations of care (Australian Government, 2021). The lack of locally developed evidence creates challenges for occupational therapists working in Australian RACFs to use evidence which recognises these institutional influences on practice. As outlined in the Australian Occupational Therapy Competency Standards (Occupational Therapy Board of Australia, 2018), there is an expectation that occupational therapists use evidence to inform their practice. The lack of Australian evidence makes it difficult for occupational therapists working in RACFs to uphold this standard. Therefore, future research should explore current practice as well as the potential of occupational therapy in Australian RACFs to support therapists to benefit the lives of Australian residents.

Australian literature, which explored occupational therapy in other practice settings or explored the contribution of other health professionals in RACFs, supports the recommendations discussed in this review. A recent study, which explored the promotion of evidence‐based occupational therapy interventions in community aged care, identified that promoting evidence‐based interventions had positive outcomes for the profession such as an increased professional identity (Culph et al., 2021). The authors claim that evidence supports occupational therapists to provide intervention that reflects their full scope of practice for the benefit of older adults residing in the community (Culph et al., 2021). This further supports our recommendation of building the evidence base of occupational therapy in Australian RACFs to support the lobbying of national associations and occupational therapists to elicit positive outcomes for Australian residents. Furthermore, Rayner et al. (2020) identified research priorities for Australian RACFs. This included exploring how to best support residents with dementia and investigation of funding due to reports of the ACFI being inadequate. These priorities were identified for a range of reasons including the reported challenge of keeping residents physically and mentally active. Occupational therapy, the profession concerned with occupational engagement, is the key health profession to respond to this challenge. Similar findings regarding the ACFI were reported in a physiotherapy paper conducted by Brett et al. (2018), who concluded that the ACFI restricts physiotherapy practice. As occupational therapists are positioned to contribute to these research priorities, it is recommended that future research explores current occupational therapy practice under the ACFI, and the potential contribution occupational therapists can have in RACFs.

This review has identified a crucial need to develop Australian literature that explores occupational therapy practice in RACFs. By building the current evidence base, this will support national associations to advocate for the profession and support occupational therapists to enrich the lives of older adults residing in RACFs. Additionally, this review reveals that other professions (Baldacchino & Bonello, 2013a; Baldacchino & Bonello, 2013b; Blackler et al., 2018) are making recommendations for future occupational therapy practice. This has the potential to influence practice to be inconsistent with the profession's unique occupational philosophy. Therefore, further research by occupational therapists in this practice setting must be conducted to build the evidence base and promote consistency between the evidence and the profession's core knowledge, skills and values. However, the ACFI may be imposing challenges for clinicians to conduct research. Therefore, academic–clinician partnerships (Stern, 2005), internal to the profession, are recommended. This strategy has been employed previously and successfully addressed barriers to conducting research, including time constraints experienced by clinical occupational therapists (Stern, 2005).

## 5 LIMITATIONS

A systematic mapping review does not include critical appraisal or comprehensive analysis of the findings of the included articles. Additionally, not all articles included in this review are exclusively Australian or set in RACFs. This creates a small risk that the extracted data may not be relevant to the research question.

## 6 IMPLICATIONS FOR PRACTICE AND FUTURE RESEARCH

This review identified a scarcity of research evidence within Australia. This includes research that exclusively explores the experiences of occupational therapists working in Australian RACFs and how occupational therapists support residents to achieve their occupational goals. There is a need for high‐quality research to explore and evaluate both current and alternative models of occupational therapy practice in Australian RACFs to demonstrate the contribution the profession can offer. Development of current scholarship will support the lobbying of national associations and support clinical occupational therapists to elicit positive outcomes for their clients.

## CONFLICT OF INTEREST

The authors have no conflict of interest to declare.

## AUTHOR CONTRIBUTION

All authors contributed to the development and approval of this systematic mapping review. This included screening the retrieved papers to identify relevant articles and completion of data analysis to promote validity. All authors contributed to the development of the manuscript and are, therefore, accountable for ensuring this manuscript answers the research question that guided this review. The study was conceptualized by the second and third authors. The first author completed the literature search. All authors screened articles, extracted data, and synthesized and interpreted results. All authors contributed to the manuscript.

## Data Availability

Data sharing is not applicable to this article as no datasets were generated or analyzed during the current study.
